# Supplementary material for: Skin graft with dermis and appendages generated in vivo by cell competition
Source: Nat Commun. 2024 Apr 29;15:3366. doi: 10.1038/s41467-024-47527-7 (PMC11058811; doi:10.1038/s41467-024-47527-7)
Supplement: Supplementary file 1 — Supplementary Information [file 41467_2024_47527_MOESM1_ESM.pdf]

Skin graft with dermis and appendages generated in vivo by cell competition

Hisato Nagano[1,2,3], Naoaki Mizuno[1,2,4 *], Hideyuki Sato[1,2], Eiji Mizutani[1,2,5], Ayaka Yanagida[1,2,6], Mayuko Kano[1,2,7], Mariko Kasai[1,2], Hiromi Yamamoto[1,2], Motoo Watanabe[1,2], Fabian Suchy[8] Hideki Masaki[1,2] and Hiromitsu Nakauchi[1,2,8, *]

Affiliations

[1]Division of Stem Cell Therapy, Center for Stem Cell Biology and Regenerative Medicine, Institute of Medical Science, University of Tokyo, 4-6-1 Shirokanedai, Minato-ku, Tokyo 108-8639, Japan

[2]Stem Cell Therapy Laboratory, Advanced Research Institute, Tokyo Medical and Dental University, 1-5-45 Yushima, Bunkyo-ku, Tokyo 113-8510, Japan

[3]Department of Plastic and Reconstructive Surgery, National Defense Medical College, 3-2 Namiki, Tokorozawa, Saitama 359-8513, Japan

[4]Department of Experimental Animal Model for Human Disease, Center for Experimental Animals, Tokyo Medical and Dental University, 1-5-45 Yushima, Bunkyo-ku, Tokyo 113-8510, Japan

[5]Laboratory of Stem Cell Therapy, Institute of Medicine, University of Tsukuba, 1-1-1 Tennodai, Tsukuba, Ibaraki 305-8577, Japan

[6]Department of Veterinary Anatomy, The University of Tokyo, Yayoi 1-1-1, Bunkyo-ku, Tokyo 113-8657, Japan

[7]Metabolism and Endocrinology, Department of Medicine, St. Marianna University School of Medicine, 2-16-1 Sugao, Miyamae-ku, Kawasaki, Kanagawa 216-8511, Japan

[8]Institute for Stem Cell Biology and Regenerative Medicine, Stanford University School of Medicine, Sanford, CA 94305, USA

Supplementary Fig. 1

a

Embryo transferred	Implantation	Live fetus	KO phenotype (+)	Biallelic loss-of-function mutations
86	48 (55.8%)	22 (25.6%)	17 (77.3%)	11 (50%)

b

No.	KO phenotype	Genotype of detactable allele	Biallelic loss-of-function mutation
1	+	Out-of-flame mutation	Yes
2	+	Out-of-flame mutation	Yes
3	+	In-flame mutation	No
4	+	Out-of-flame mutation	Yes
5	+	Out-of-flame mutation	Yes
6	-	In-flame mutation, Out-of-flame mutation	No
7	-	Out-of-flame mutation, WT allele	No
8	-	In-flame mutation, Out-of-flame mutation	No
9	-	Out-of-flame mutation, WT allele	No
10	+	Large deletion	Yes
11	+	Large deletion	Yes
12	+	In-flame mutation, Out-of-flame mutation	No
13	+	Out-of-flame mutation	Yes
14	+	In-flame mutation, Out-of-flame mutation	No
15	+	In-flame mutation, Out-of-flame mutation	No
16	+	Large deletion	Yes
17	+	In-flame mutation, Out-of-flame mutation	No
18	-	Out-of-flame mutation, WT allele	No
19	+	Out-of-flame mutation	Yes
20	+	Out-of-flame mutation	Yes
21	+	In-flame mutation, Out-of-flame mutation	No
22	+	Out-of-flame mutation	Yes

c

Embryo number	Total efficiency	In frame mutation	Out of frame mutation	Allele frequency	WT allele	R^2
#1	94.5%	0%	86.2%	-7, 7.8%; -41, 76.6%	0%	0.94
#2	97%	0%	97%	-2, 34.5%; -7, 62.7%	0%	0.97
#3	93%	0%	93%	-2, 9.8%; -5, 23%; -7, 29.5%; -20, 29.2%	0%	0.92

d

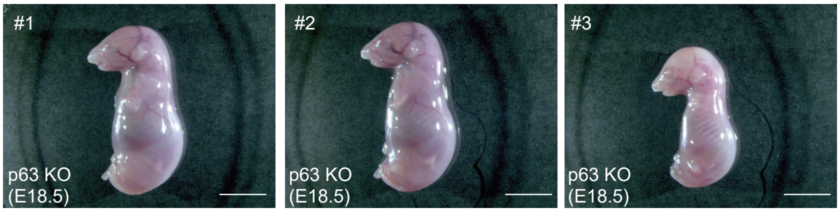

e

Embryo transferred	Live fetus	non-chimera	chimera	Biallelic loss-of-function mutations
79	21 (26.6%)	13 (16.5%)	8 (10.1%)	7

f

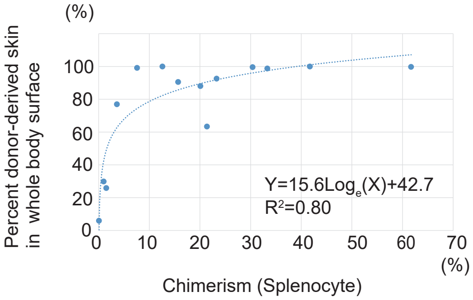

Supplementary Fig. 1: The total skin area in p63 knockout chimera tends to expand in proportion to global chimerism, and no SER remained in the generated skin.

a, The developmental rate and genotypes of p63 knockout embryo.

Loss-of-function mutations were defined as out-of-frame mutations and large deletions. The embryo with the p63 knockout phenotype, such as thin transparent skin on the whole body, was counted as knockout (KO) phenotype (+) regardless of the genotypes.

b, Phenotype and genotype of live fetuses.

c, Representative detailed genotypes of p63 knockout embryos.

d, Macroscopic images of p63 knockout embryos with biallelic mutation (out/out). Scale bars, 5 mm.

e, The developmental rate and genotypes of p63 knockout chimeras.

f, Correlation between the body surface coverage by donor-derived keratinocytes and global chimerism in p63 knockout chimera.

Source data are provided with this paper.

Supplementary Fig. 2

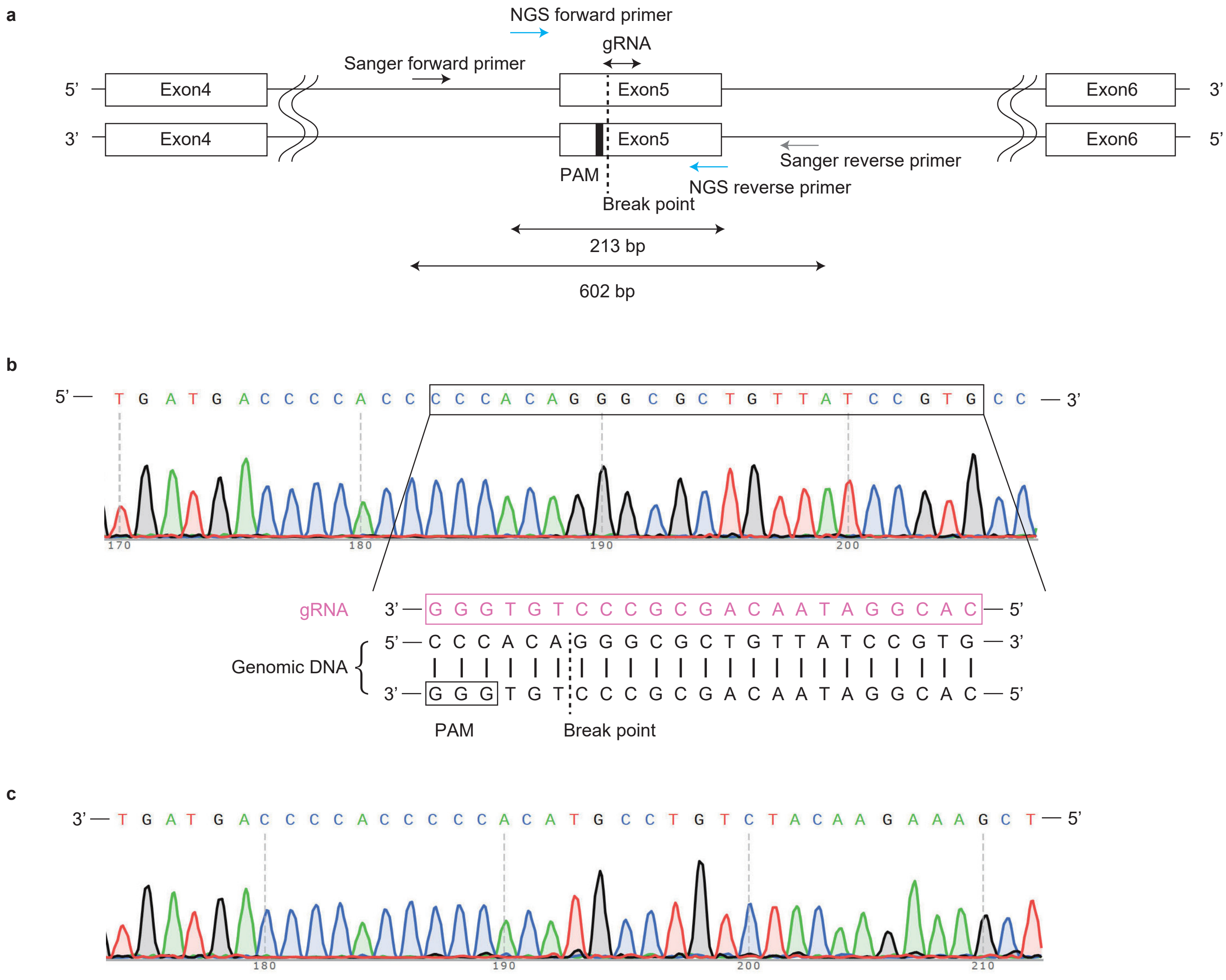

d

	Detected allele	
	Major	Minor
Sanger sequencing	20-bp deletions (96.1%)	7-bp deletions (2.6%)
NGS-based sequencing	20-bp deletions (94.6%)	7-bp deletions (5.4%)

Supplementary Fig. 2: Genotype determination by TIDE analysis and NGS-based genotyping

a, Designing of gRNA for CRISPR-Cas9 and primers for genotyping. Black arrows, PCR forward and Sanger sequencing primer; gray arrow, PCR reverse primer; blue arrows. NGS primers.

b-d, Representative of embryo genotype.

b, Sanger sequencing of wild type embryo. gRNA (magenta box). Break point (dotted line). GGG in the black box indicated protospacer adjacent motif (PAM).

c, Sanger sequencing of p63 knockout embyro.

d, TIDE analysis of Sanger sequencing and NGS-based genotyping of the same p63 mutant embryo. The embryo was same as c.

Supplementary Fig. 3

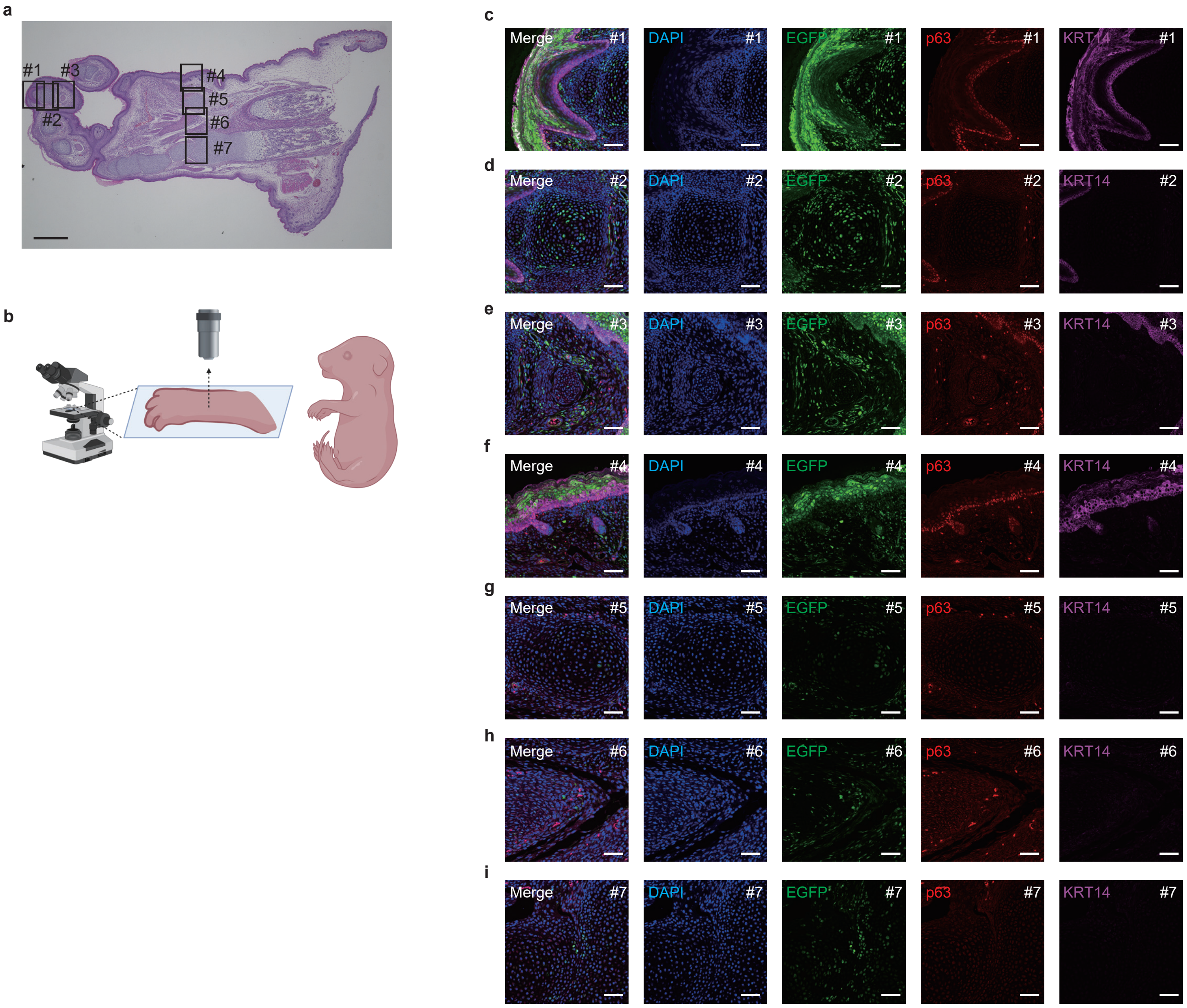

Supplementary Fig. 3: The limb composed of donor PSC-derived epidermis on chimeric dermis and deep tissues in p63 KO chimera.

a, Histological staining of the forelimb of p63 knockout chimera skin. Scale bar, 500 μm. Additional immunofluorescence staining was performed in the black box (#1-7)

b, Direction of sample sectioning. The sample was horizontally sectioned to the palm. Created with BioRender.com.

c-i, Immunofluorescence staining of the forelimb of p63 knockout chimera skin. Scale bar, DAPI, nuclear; EGFP, the label of donor cell; p63, stem cell marker; KRT14, mature keratinocyte in the basal layer. Scale bars, 50 μm.

Supplementary Fig. 4

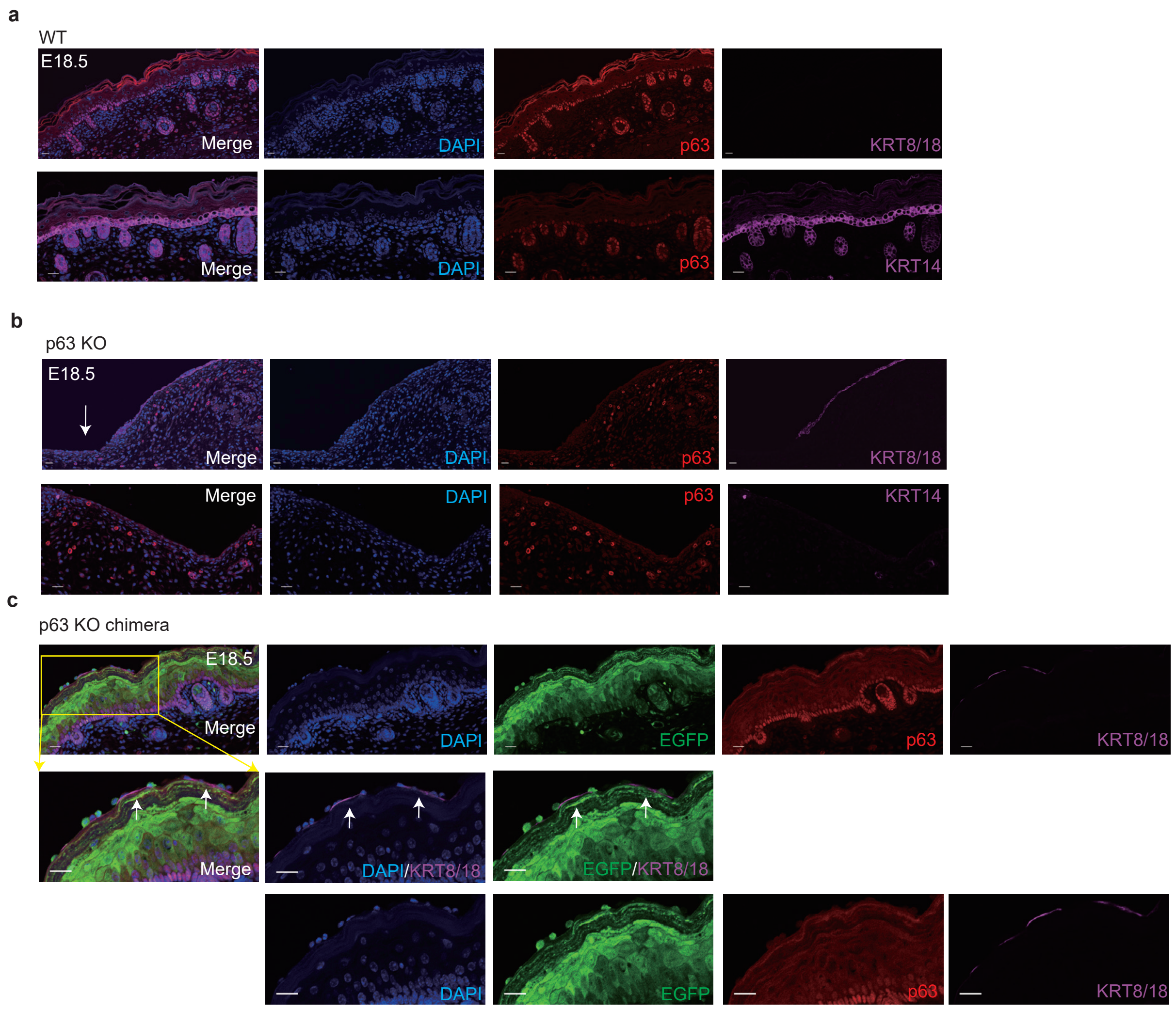

Supplementary Fig. 4: SERs express unmatured keratinocyte marker KRT8/18 in p63 KO chimeras at E18.5.

a, Immunofluorescence staining of WT embryos' skin at E18.5. Scale bars, 20 μm.

b, Immunofluorescence staining of p63 knockout embryos' skin at E18.5. Scale bars, 20 μm. Some SER was detached (white arrows).

c, Immunofluorescence staining of p63 knockout chimera skin at E18.5. Scale bars, 20 μm. The white arrows indicate SERs.

Supplementary Fig. 5

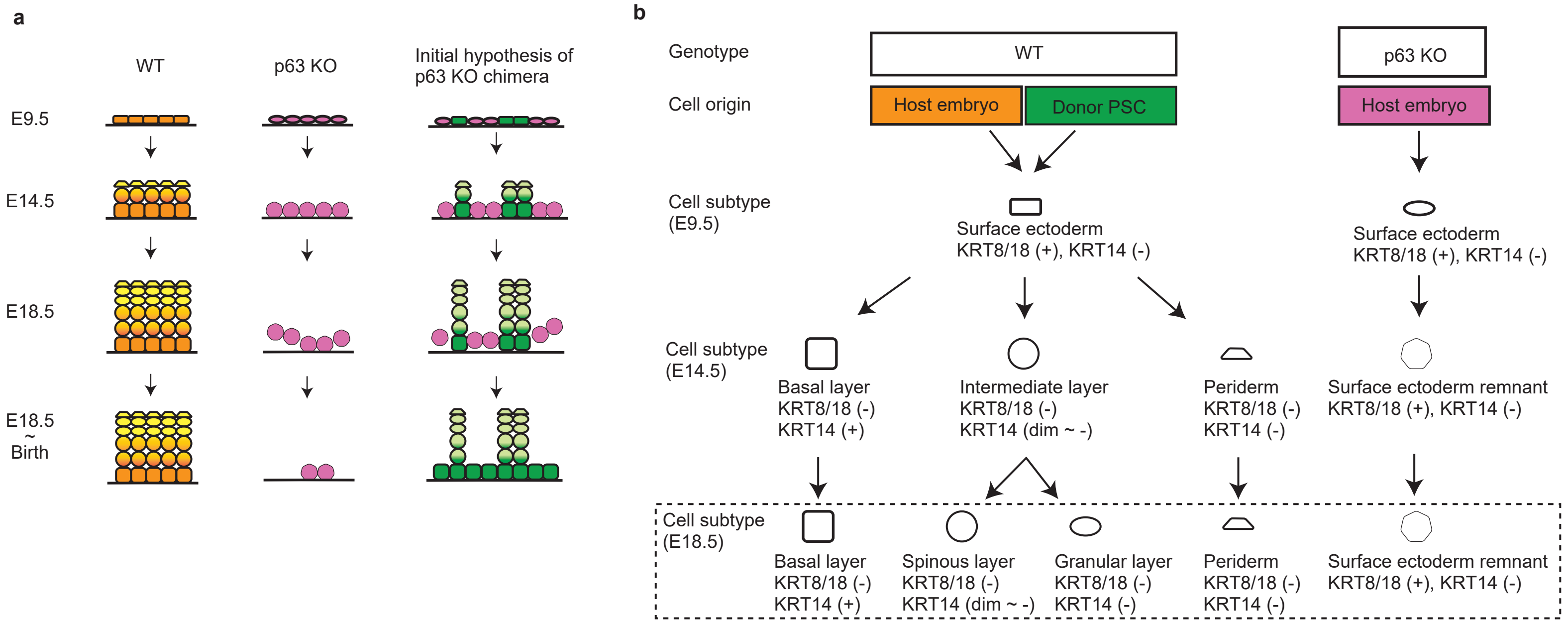

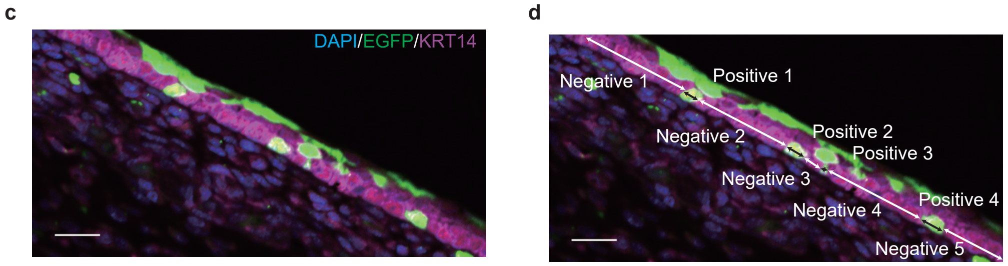

e

Region	Length	Frequency
EGFP (+)	243	Fp=0.11
EGFP (-)	2008	Fn=0.89
Total	2251	1

$$Fp = \frac{\Sigma Lp}{\Sigma Lp + \Sigma Ln} = \frac{243}{243 + 2008} = 0.11$$

$$Fn = \frac{\Sigma Ln}{\Sigma Lp + \Sigma Ln} = \frac{2008}{243 + 2008} = 0.89$$

f

Region	Length	Probability (=P)	-logP
Negative 1	729	6.8E-37	36.2
Positive 1	77	3.6E-75	75.2
Negative 2	417	2.1E-21	20.7
Positive 2	85	6.7E-83	82.2
Negative 3	87	4.8E-5	4.3
Positive 3	23	5.8E-23	22.2
Negative 4	446	7.5E-23	22.1
Positive 4	58	8.5E-57	56.1
Negative 5	329	4.8E-17	16.3

$Probability = (Fn)^{Length}$ $Probability = (Fp)^{Length}$

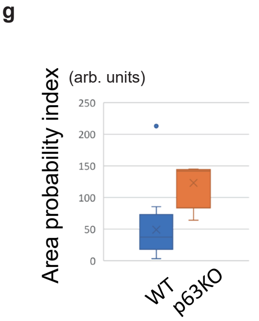

Supplementary Fig. 5: Hypothesis of skin development in p63 knockout chimera and quantitative evaluation of the mosaic distribution.

a, Skin development in WT embryos (left column), p63 KO embryos (center column), and the initial hypothesis of skin development in p63 KO chimeras (right column).

b, Cell subtypes and cytokeratin expression patterns for each genotype and cell origin.

c-f, Based on immunofluorescence staining images of p63 knockout chimera at E14.5 (c), pixels of contiguous adjacent regions between cells derived from donor PSCs ($EGFP^+$) and host embryos ($EGFP^-$) were measured (d). The EGFP-positive and EGFP-negative pixel frequency were calculated as Fp and Fn, respectively (e). The probability of each region was calculated as Fn^{Length} of each region (f). The area probability index was -log10P.

g, Comparison of the probability of consecutive possible adjacent donor PSC based on chimerism in the epidermis of WT chimera and p63 knockout chimera. Source data are provided with this paper.

Supplementary Fig. 6

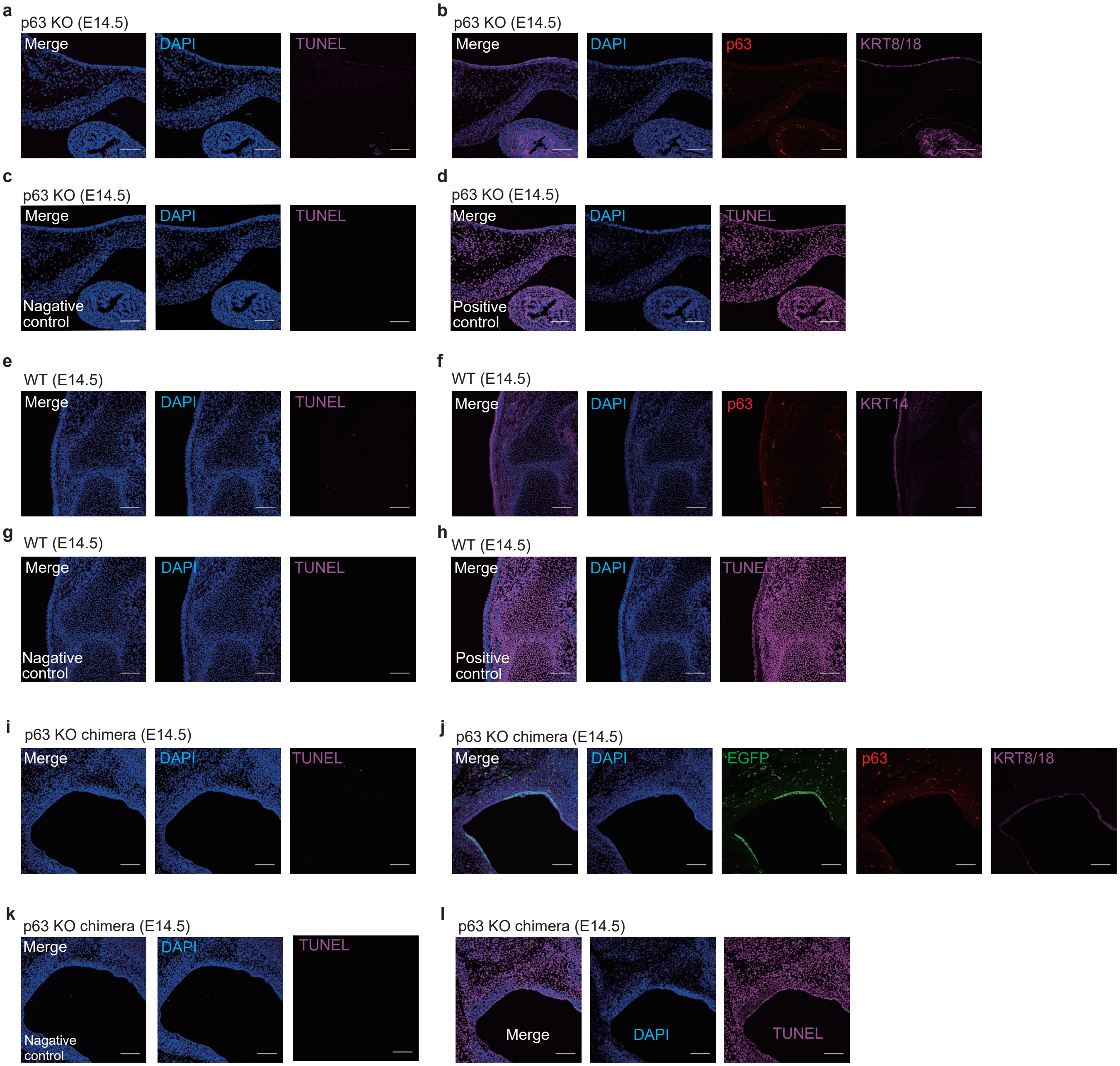

Supplementary Fig. 6: Evaluation of apoptosis by TUNEL staining.

a-d, p63 knockout embryos (E14.5), e-h, WT embryos, i-l, p63 knockout chimera. Serial sections. Scale bars, 100 μm.

a, e, i, TUNEL staining. There was no significant difference in TUNEL-positive cells among p63 knockout embryos, WT embryos, or p63 knockout chimera.

b, f, j, Immunofluorescence staining for the epidermal cytokeratins.

c, g, k, Negative control without TUNEL staining.

d, h, l, Positive control with a double-strand break following DNase treatment.

Supplementary Fig. 7

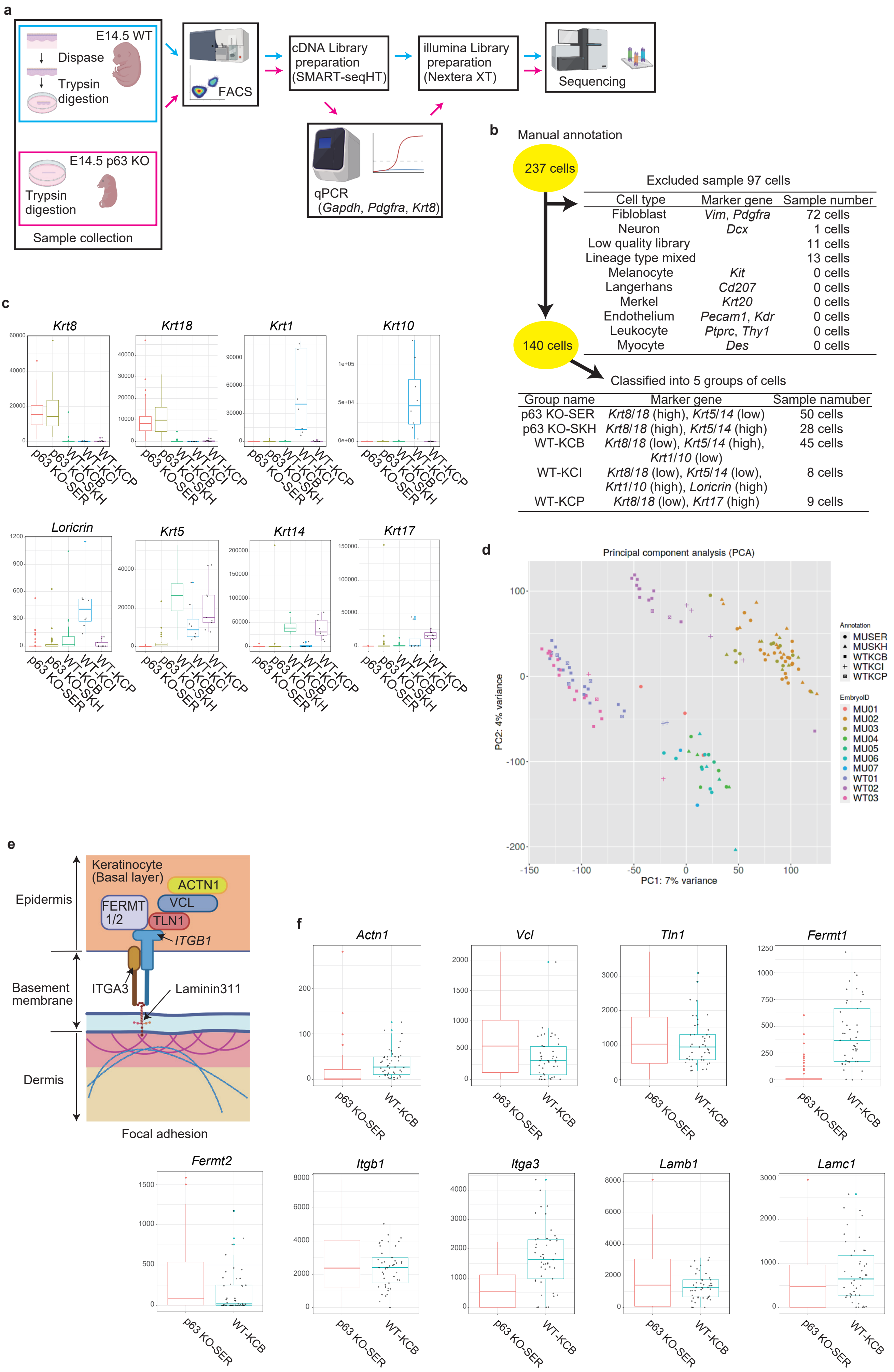

Supplementary Fig. 7: Single-cell RNA-seq of keratinocytes from wild-type and p63 knockout mice.

a, Workflow of the single-cell RNA-seq. Created with BioRender.com.

b, Manual annotation of the cell subtypes. p63KO-SER, surface ectoderm remnant in p63KO embryos; p63KO-SKH, surface ectoderm remnant with the mature keratinocytes marker in p63 KO embryos; WT-KCB, keratinocytes in basal layer of WT embryos; WT-KCI, keratinocytes in intermediate layer of WT embryos; WT-KCP, keratinocytes in periderm of WT embryos.

c, The marker gene expression. Source data are provided in the Supplementary Data 1.

d, Principal component analysis (PCA). Circle, p63 KO-SER; triangle, p63 KO-SKH; box, WT-KCB, cross, WT-KCI; cross box, WT-KCP. Symbol colors indicate embryo ID. Source data are provided with this paper.

e, Schematics of focal adhesion. Created with BioRender.com.

f, Comparison of gene expression between p63 knockout-SER (Surface ectoderm remnant) and WT-KCB (keratinocyte in basal layer). Source data are provided in the Supplementary Data 1.

Supplementary Fig. 8

a

Group	Symbol color	Sample size	Embryos	Donor cells	Epidermis	Dermis	Recipient
Semi-autologous		4	p63 KO	C57BL/6	C57BL/6	Chimeric	C57BL/6-Tg
Allogeneic		4	WT	C57BL/6	Chimeric	Chimeric	C57BL/6-Tg

b

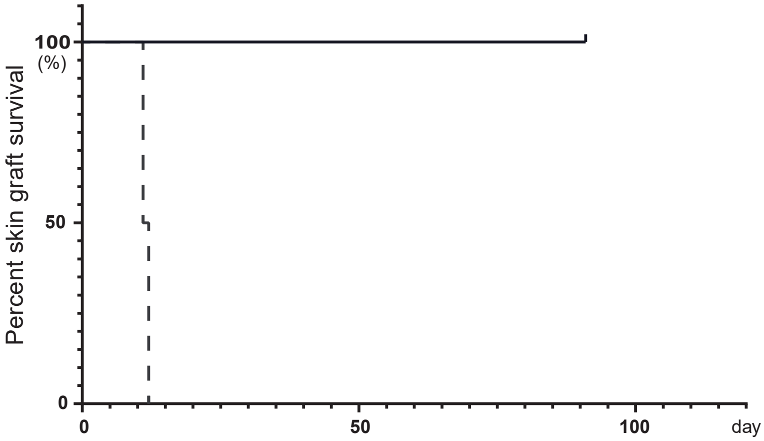

c

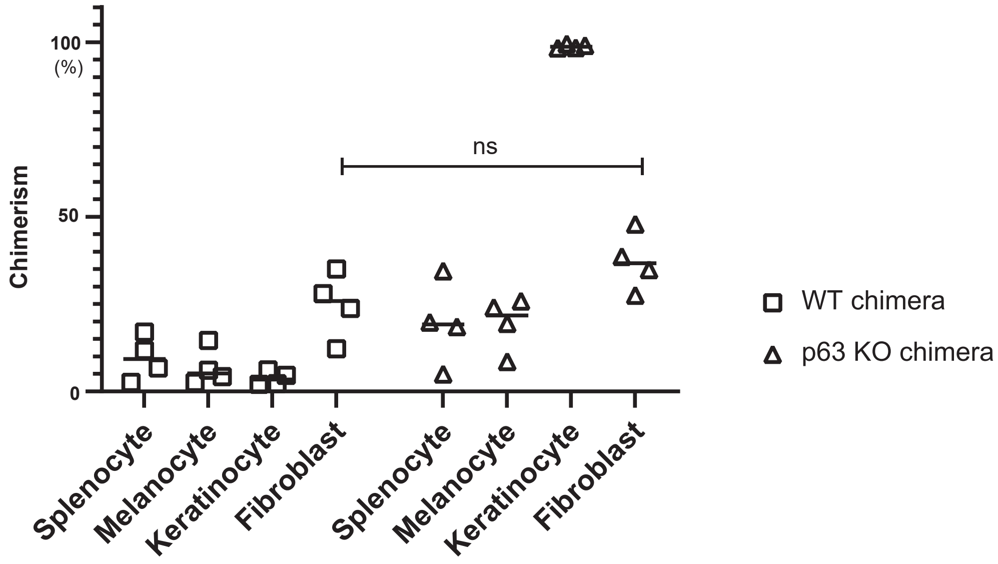

Supplementary Fig. 8: A subgroup analysis of skin grafting within similar dermal chimerism.

a, The details of extracted experimental groups.

b, Graft survival curves. Solid line, semi-autologous grafting; Dotted line, allogenic.

c, Chimerism of experimental groups. Statistical analysis was performed using Mann-Whitney tests.

Source data are provided with this paper.

Supplementary Table 1

	Gene	p value	Significance
Hemidesmosome	*Krt14*	4.02618E-43	Yes
Hemidesmosome	*Krt5*	9.0936E-181	Yes
Hemidesmosome	*Dst*	4.12851E-09	Yes
Hemidesmosome	*Plec*	0.385757317	No
Hemidesmosome	*Itga6*	0.000174963	Yes
Hemidesmosome	*Itgb4*	3.32996E-07	Yes
Hemidesmosome	*Col17a1*	1.9899E-168	Yes
Hemidesmosome	*Lama3*	6.22533E-08	Yes
Hemidesmosome	*Lamb3*	1.26053E-07	Yes
Hemidesmosome	*Lamc2*	0.196635582	No

	Gene	p value	Significance
Focal adhesion	*Actn1*	0.237778455	No
Focal adhesion	*Vcl*	0.060073518	No
Focal adhesion	*Tln1*	0.480629924	No
Focal adhesion	*Fermt1*	4.16831E-06	Yes
Focal adhesion	*Fermt2*	0.128404949	No
Focal adhesion	*Itga3*	0.001495203	Yes
Focal adhesion	*Itgb1*	0.458308876	No
Focal adhesion	*Lama3*	6.22533E-08	Yes
Focal adhesion	*Lamb1*	0.245513863	No
Focal adhesion	*Lamc1*	0.420828665	No
