# Supplementary material for: Skin graft with dermis and appendages generated in vivo by cell competition
Source: Nat Commun. 2024 Apr 29;15:3366. doi: 10.1038/s41467-024-47527-7 (PMC11058811; doi:10.1038/s41467-024-47527-7)
Supplement: Supplementary file 3 — Description of Additional Supplementary Files [file 41467_2024_47527_MOESM3_ESM.pdf]

Description of Additional Supplementary Files

Supplementary Data 1: Normalized gene expression matrix and DEG analysis of samples-of-interest with annotations.

Supplementary Data 2: Primer information for NGS based genotyping.
