# Supplementary material for: Skin graft with dermis and appendages generated in vivo by cell competition
Source: Nat Commun. 2024 Apr 29;15:3366. doi: 10.1038/s41467-024-47527-7 (PMC11058811; doi:10.1038/s41467-024-47527-7)
Supplement: Supplementary file 6 — Reporting Summary [file 41467_2024_47527_MOESM6_ESM.pdf]

nature portfolio

Corresponding author(s): Naoaki Mizuno, Hiromitsu Nakauchi.

Last updated by author(s): Feb 21, 2024

Reporting Summary

Nature Portfolio wishes to improve the reproducibility of the work that we publish. This form provides structure for consistency and transparency in reporting. For further information on Nature Portfolio policies, see our Editorial Policies and the Editorial Policy Checklist.

Statistics

For all statistical analyses, confirm that the following items are present in the figure legend, table legend, main text, or Methods section.

n/a	Confirmed	
☐	☒	The exact sample size (*n*) for each experimental group/condition, given as a discrete number and unit of measurement
☐	☒	A statement on whether measurements were taken from distinct samples or whether the same sample was measured repeatedly
☐	☒	The statistical test(s) used AND whether they are one- or two-sided<br>*Only common tests should be described solely by name; describe more complex techniques in the Methods section.*
☒	☐	A description of all covariates tested
☒	☐	A description of any assumptions or corrections, such as tests of normality and adjustment for multiple comparisons
☐	☒	A full description of the statistical parameters including central tendency (e.g. means) or other basic estimates (e.g. regression coefficient) AND variation (e.g. standard deviation) or associated estimates of uncertainty (e.g. confidence intervals)
☐	☒	For null hypothesis testing, the test statistic (e.g. *F*, *t*, *r*) with confidence intervals, effect sizes, degrees of freedom and *P* value noted<br>*Give P values as exact values whenever suitable.*
☒	☐	For Bayesian analysis, information on the choice of priors and Markov chain Monte Carlo settings
☒	☐	For hierarchical and complex designs, identification of the appropriate level for tests and full reporting of outcomes
☒	☐	Estimates of effect sizes (e.g. Cohen's *d*, Pearson's *r*), indicating how they were calculated

Our web collection on statistics for biologists contains articles on many of the points above.

Software and code

Policy information about availability of computer code

Data collection	No software for data collection was used.
Data analysis	We used FlowJo (BD biosciences, New Jersey, USA) and GraphPad Prism, v.8.4.3 for data analysis.

For manuscripts utilizing custom algorithms or software that are central to the research but not yet described in published literature, software must be made available to editors and reviewers. We strongly encourage code deposition in a community repository (e.g. GitHub). See the Nature Portfolio guidelines for submitting code & software for further information.

Data

Policy information about availability of data

All manuscripts must include a data availability statement. This statement should provide the following information, where applicable:

- Accession codes, unique identifiers, or web links for publicly available datasets
- A description of any restrictions on data availability
- For clinical datasets or third party data, please ensure that the statement adheres to our policy

We mapped the data to mouse reference genome mm10 by HISAT2 (Galaxy Version2.1.0+galaxy5) on Galaxy (version20.05) and counted by Feature Counts (Galaxy Version 1.6.4+galaxy2) based on the GTF file of Ensemble, version-release.97. Transcript integrity number (TIN) and Gene Body Coverage were analyzed by RSeQC (Galaxy Version 2.6.4.1 and Galaxy Version 2.6.4.3) to exclude low-quality samples. The count was normalized by DESeq2 (version1.38.2) and analyzed for differentially expressed genes (DEGs). The P-value was calculated using a two-sided Wald test. We visualized the data using iDEP (version0.96) or R (version3.6.0 and

4.2.2) ggplot2.
The scRNA-seq dataset was deposited at SRA: BioProject PRJNA1056324, GEO accession GSE252023.

Research involving human participants, their data, or biological material

Policy information about studies with human participants or human data. See also policy information about sex, gender (identity/presentation), and sexual orientation and race, ethnicity and racism.

Reporting on sex and gender	No human participants nor human data in this manuscript.
Reporting on race, ethnicity, or other socially relevant groupings	No human participants nor human data in this manuscript.
Population characteristics	No human participants nor human data in this manuscript.
Recruitment	No human participants nor human data in this manuscript.
Ethics oversight	No human participants nor human data in this manuscript.

Note that full information on the approval of the study protocol must also be provided in the manuscript.

Field-specific reporting

Please select the one below that is the best fit for your research. If you are not sure, read the appropriate sections before making your selection.

☒ Life sciences ☐ Behavioural & social sciences ☐ Ecological, evolutionary & environmental sciences

For a reference copy of the document with all sections, see nature.com/documents/nr-reporting-summary-flat.pdf

Life sciences study design

All studies must disclose on these points even when the disclosure is negative.

Sample size	No sample size calculation is performed. The sample size was determined based on test samples' availability and the animal welfare. They are enough large empirically for the statistical analysis.
Data exclusions	Some datasets in the single cell RNA-seq analysis are excluded because of low quality estimated by RSeQC package.
Replication	All experiments are performed at least three independent trials. All attempts at replication were successful.
Randomization	Skin graft obtained from the same individual was divided into multiple portions and transplanted into the experimental and control groups, respectively. So, randomization is not performed in this experiment.
Blinding	We are not blinded to perform sample collection and skin grafting experiment because the difference between WT embryo and p63 knockout embryo is clearly evident with the naked eye. Data collection for all automated experiments (e.g. flow cytometry, sequencing, etc) and data analysis was based on appropriate controls.

Reporting for specific materials, systems and methods

We require information from authors about some types of materials, experimental systems and methods used in many studies. Here, indicate whether each material, system or method listed is relevant to your study. If you are not sure if a list item applies to your research, read the appropriate section before selecting a response.

Materials & experimental systems

n/a	Involved in the study	
☐	☒	Antibodies
☐	☒	Eukaryotic cell lines
☒	☐	Palaeontology and archaeology
☐	☒	Animals and other organisms
☒	☐	Clinical data
☒	☐	Dual use research of concern
☒	☐	Plants

Methods

n/a	Involved in the study	
☒	☐	ChIP-seq
☐	☒	Flow cytometry
☒	☐	MRI-based neuroimaging

Antibodies

Antibodies used	For flow cytometry, anti-CD45 (clone 30-F11, Biolegend), anti-CD117 (clone 2B8, eBioscience), and anti-CD49f (clone GoH3, eBioscience).<br>For IHC, mouse anti-p63 (1:100, clone D-9, sc-25268, Santa Cruz), rabbit anti-Cytokeratin 1 (1:200, polyclonal, ab93652, Abcam), rabbit anti-Cytokeratin 5 (1:200, clone EP1601Y, ab52635, Abcam), rabbit anti-Cytokeratin 10 (1:200, clone EP1607IHCY, ab76318, Abcam), ,rabbit anti-Cytokeratin 14 (1:200, clone EPR17350, ab181595, Abcam), rabbit anti-Cytokeratin 8/18 (1:200, clone EP1628Y, ab53280, Abcam), rabbit anti-Loricrin (1:200, polyclonal, ab85679, Abcam), rabbit anti-Involucrin (1:200, clone EPR13054, ab181980, Abcam) and goat anti-GFP (1:200, polyclonal, ab6673, Abcam). Secondary antibodies: Donkey anti-goat IgG Alexa Fluor 488 (1:1000, A11055: Invitrogen), Donkey anti-mouse IgG Alexa Fluor 568 (1:1000, A10037: Invitrogen), Donkey anti-rabbit IgG Alexa Fluor 568 (1:1000, A10042: Invitrogen) and Donkey anti-rabbit IgG Alexa Fluor 647 (1:1000, A31573: Invitrogen).
Validation	Antibody reactivities are validated by the supplier as follows.<br>p63 (mouse, rat, human)<br>Cytokeratin 1 (human)<br>Cytokeratin 5 (mouse, rat, human)<br>Cytokeratin 10 (mouse, rat, human)<br>Cytokeratin 14 (mouse, rat, human)<br>Cytokeratin 8/18 (mouse, rat, human)<br>Loricrin (mouse, human)<br>Involucrin (rat, Human)<br>GFP (Species independent)<br><br>Additional validation data for the antibodies are available from the following websites:<br>https://www.scbt.com/p/p63-antibody-d-9<br>https://www.abcam.com/en-hk/products/primary-antibodies/cytokeratin-1-antibody-ab93652#<br>https://www.abcam.com/en-sg/products/primary-antibodies/cytokeratin-5-antibody-ep1601y-cytoskeleton-marker-ab52635<br>https://www.abcam.com/en-mc/products/primary-antibodies/cytokeratin-10-antibody-ep1607ihcy-cytoskeleton-marker-ab76318<br>https://www.abcam.com/en-at/products/primary-antibodies/cytokeratin-14-antibody-epr17350-cytoskeleton-marker-ab181595<br>https://www.abcam.com/en-al/products/primary-antibodies/cytokeratin-8-antibody-ep1628y-cytoskeleton-marker-ab53280<br>https://www.abcam.com/en-ug/products/primary-antibodies/loricrin-antibody-ab85679<br>https://www.abcam.com/en-by/products/primary-antibodies/involucrin-antibody-epr13054-ab181980<br>https://www.abcam.com/en-it/products/primary-antibodies/gfp-antibody-ab6673

Eukaryotic cell lines

Policy information about cell lines and Sex and Gender in Research

Cell line source(s)	HaCaT, human immortalized keratinocytes, male, were purchased from Cell Line Service.
Authentication	HaCaT is distributed by German Cancer Research Center (DKFZ) and was purchased from Cell Line Service (product #300493). It is not listed on the known Misidentified Cell Lines list version 12 of the International Cell Line Authentication Committee.
Mycoplasma contamination	Mycoplasma contamination is declined by MycoAlert (Lonza).
Commonly misidentified lines<br>(See ICLAC register)	Not listed on the known Misidentified Cell Lines list version 12 of the International Cell Line Authentication Committee.

Animals and other research organisms

Policy information about studies involving animals; ARRIVE guidelines recommended for reporting animal research, and Sex and Gender in Research

Laboratory animals	C57BL/6N, ICR, C57BL/6N-Tg (CAG-EGFP), and DBA/2 mice were purchased from SLC Japan (Shizuoka, Japan). They are sacrificed and donated for experiments from E9.5 embryonic day to 20 weeks.<br>All mice were reared under the prescribed conditions with a 12-h dark (PM 20:00- AM 8:00)/12-h light (AM 8:00- PM 20:00) cycle at 23 ± 2 °C and humidity of 50 ± 10%.
Wild animals	No wild animals are used.
Reporting on sex	Animal sex is not biased. Both male and females are used. However, it is impossible to count number of each sex, because we use embryos and chimeras.
Field-collected samples	No field-collected samples.
Ethics oversight	All experiments were performed in accordance with the animal care and use committee guidelines of the Institute of Medical Science, the University of Tokyo (Permission No. PA21-16, PA21-47).

Note that full information on the approval of the study protocol must also be provided in the manuscript.

Plants

Seed stocks	No seeds stocks and other plant material are used
Novel plant genotypes	No seeds stocks and other plant material are used
Authentication	No seeds stocks and other plant material are used

Flow Cytometry

Plots

Confirm that:

☒ The axis labels state the marker and fluorochrome used (e.g. CD4-FITC).

☒ The axis scales are clearly visible. Include numbers along axes only for bottom left plot of group (a 'group' is an analysis of identical markers).

☒ All plots are contour plots with outliers or pseudocolor plots.

☒ A numerical value for number of cells or percentage (with statistics) is provided.

Methodology

Sample preparation	The skin was harvested from p63 knockout chimera and WT-chimera and digested in CnT-07 medium supplemented with 25 U/ml dispase (Gibco, Life Technologies, Carlsbad, CA) at 4 °C, 12-24 hours. Epidermis isolated from the skin was digested with accutase (Innovative Cell Technologies, California, USA) to prepare single-cell suspensions.<br>Spleens were dissociated into single cells by pipetting, hemolyzed in ACK buffer, and stained with anti-CD45 antibody for 30 min.
Instrument	CytoFLEX S (B75442, BECKMAN COULTER); FACSAria II (BD biosciences); FACSAria III (BD biosciences).
Software	FlowJo software (BD biosciences)
Cell population abundance	Each sample was stained with Propidium iodide (PI) (Sigma-Aldrich, St. Louis, MO) or 4',6-diamidino-2-phenylindole (DAPI) (Invitrogen, Carlsbad, CA). Samples were stained with anti-CD45 (clone 30-F11, Biolegend), anti-CD117 (clone 2B8, eBioscience), and anti-CD49f (clone GoH3, eBioscience) antibodies. Detailed information about gating and population frequency is shown in Figure 1d. In brief, PI-negative CD45+ fraction is about 2-3%, PI-negative CD45- CD117+ fraction is 6%, and PI-negative CD117-CD49f+ fraction is 89-91%.
Gating strategy	FSC/SSC gating is defined empirically to exclude debris and dead cells. For all other channels, signals are compensated optimally according single antibody staining and the thresholds are defined according to unstained controls.

☒ Tick this box to confirm that a figure exemplifying the gating strategy is provided in the Supplementary Information.
